# The use of smartphones to influence lifestyle changes in overweight and obese youth with congenital heart disease: a single-arm study: Pilot and feasibility study protocol: Smart Heart Trial

**DOI:** 10.1186/s40814-017-0207-y

**Published:** 2017-11-15

**Authors:** Meghan Rombeek, Stefanie De Jesus, Luis Altamirano-Diaz, Eva Welisch, Harry Prapavessis, Jamie A. Seabrook, Kambiz Norozi

**Affiliations:** 1 Department of Paediatrics, Western University, London Health Sciences Centre, London, Canada; 2 School of Kinesiology, Western University, London, Canada; 3 Brescia University College, Western University, London, Canada; 4 Department of Paediatric Cardiology and Intensive Care Medicine, Medical School Hannover, Hanover, Germany; 5 Department of Paediatric Cardiology and Intensive Care Medicine, University of Goettingen, Goettingen, Germany; 6 Children’s Health Research Institute, London, Canada; 7 Lawson Health Research Institute, London, Canada; 8 Department of Paediatrics, Division of Paediatric Cardiology, Western University, 800 Commissioners Rd E, PO Box 5010, London, ON N6A 5W9 Canada

**Keywords:** Congenital heart disease, Smart mobile technology, Children, Adolescents, Lifestyle counseling, Nutrition, Physical activity

## Abstract

**Background:**

Both obesity and congenital heart disease (CHD) are risk factors for the long-term cardiovascular health of children and adolescents. The addition of smart mobile technology to conventional lifestyle counseling for weight management offers great potential to appeal to technologically literate youth and can address a large geographical area with minimal burden to participants. This pilot study seeks to examine the influence of a 1-year lifestyle intervention on nutrition and physical activity-related health outcomes in overweight or obese children and adolescents with CHD.

**Methods:**

This is a pilot and feasibility study which utilizes a single-arm, prospective design with a goal to recruit 40 overweight and obese patients. The feasibility metrics will evaluate the integrity of the study protocol, data collection and questionnaires, recruitment and consent, and acceptability of the intervention protocol and primary outcome measures. The primary clinical outcome metrics are anthropometry, body composition, and cardiorespiratory exercise capacity. The secondary clinical metrics include quality of life, nutrition and physical activity behavior, lung and muscle function, and cardio-metabolic risk factors. Outcomes are assessed at baseline, 6 months, and 1 year. To date, a total of 36 children and youth (11 girls), aged 7–17 years (mean = 14.4 years), have commenced the intervention. Recruitment for the study was initiated in June 2012 and is currently ongoing.

**Discussion:**

The information provided in this paper is intended to help researchers and health professionals with the development and evaluation of similar lifestyle intervention programs. Since the application of smartphones to pediatric cardiac health and obesity management is a novel approach, and continued research in this area is warranted, this paper may serve as a foundation for further exploration of this health frontier and inform the development of a broader strategy for obesity management in pediatric cardiology.

**Trial registration:**

This pilot study was retrospectively registered at the www.ClinicalTrials.gov registry as NCT02980393 in November 2016, with the study commencing in May 2012. Study protocol version 15OCT2014.

**Electronic supplementary material:**

The online version of this article (10.1186/s40814-017-0207-y) contains supplementary material, which is available to authorized users.

## Background

Both obesity and congenital heart disease (CHD) are important risk factors for the long-term cardiovascular health of children and adolescents. With an overall incidence of approximately 1% of live births, CHD ranks among the most common birth diseases [1]. Moreover, the prevalence of CHD in older populations is on the rise, and health professionals must increasingly manage CHD in the context of complex, acquired health conditions [2, 3]. Currently, about one third of children in Canada and the USA are overweight or obese [4], and research suggests that the prevalence of overweight and obesity between CHD children and healthy children does not differ significantly [5, 6].

The numerous cardiovascular risks and physical and psychosocial health consequences of childhood obesity are well reviewed [7, 8]. Of particular relevance to this paper are the accelerated atherosclerosis, endothelial dysfunction, cardiovascular structural abnormalities, dyslipidemia, insulin resistance, and hypertension. While evidence is only emerging, children with CHD and weight problems appear more likely to exhibit additional cardiovascular risk factors compared to normal weight controls [5, 9]. This is of great concern considering the increasing prevalence of metabolic syndrome in children and adolescents [10, 11].

Changes in nutrition and physical activity, including sedentary behavior, can impact both weight status and cardiovascular disease risk [12]. A structured lifestyle intervention for overweight and obese children with CHD has the potential to diminish cardiovascular health and metabolic syndrome risk factors by improving nutrition, physical fitness, body composition, and related health outcomes. However, conventional pediatric lifestyle intervention programs struggle with barriers to their success, such as high attrition rates and therapeutic non-compliance [13].

Mobile phones may address some of the inherent challenges that plague structured lifestyle interventions. First, mobile phones enable program staff to engage with young participants in their home environments across a large geographical area, with minimal burden to participants and their supporting families. Smartphones may also appeal to a more technologically savvy generation as an additional tool to sustain motivation and engagement in a program. Finally, smart mobile technology offers advantages to program staff through access to a vast array of applications and tools to deliver program content, interact with participants, and monitor participant engagement and progress. Overall, mobile devices offer promising results for weight loss and health behaviors, but their application has not been explored in an overweight, pediatric, CHD population [14–16].

The objective of this pilot study is to examine the feasibility and impact of a 1-year, structured lifestyle intervention program in overweight and obese CHD children and adolescents using smartphones in Southwestern Ontario, Canada. The feasibility of the study will be evaluated for development of a larger study according to published recommendations [17, 18]. The integrity of the study protocol, data collection and questionnaires, recruitment and consent, and acceptability of the intervention protocol and outcome measures will all be assessed and compared to previously determine thresholds where appropriate.

## Methods/design

### Research design

This study utilizes a single-arm, prospective design. Our goal is to recruit 40 overweight and obese patients (BMI for age > 85% WHO Growth Charts for Canada) between 7 and 17 years of age, who have an operated or non-operated CHD and reside in Southwestern Ontario. The underlying diagnoses include heart defects of mild and modest complexity, including atrial, ventricular, and atrio-ventricular septal defects, Tetralogy of Fallot, and coarctation of the aorta.

Eligible candidates are selected during patient visits and through chart review by a network of cardiologists at the London Health Sciences Centre (LHSC). Questions to confirm eligibility are also posed during recruitment by telephone. Consenting individuals undergo a physical assessment and review of medications and comorbidities during the first stage of their hospital visit for baseline measures. Incentives for participation include hospital parking, lunch vouchers, and reimbursement of transportation costs.

Exclusion criteria are inability to comply with research testing or intervention components due to mental and/or physical disabilities, medications or comorbidities affecting weight or metabolic condition, and involvement in any concurrent lifestyle intervention program. Parents and guardians are encouraged to be actively involved in their child’s program and to include all family members in making healthy lifestyle changes.

Feasibility of the study will be assessed by evaluating the protocol in several different areas. The integrity of the study protocol will be examined to determine the adequacy of the inclusion/exclusion criteria, to make sure all equipment is functional and provide the desired outcome data, and staff are properly trained to administer and assess the intervention as well as the overall logistics and flow of the protocol. Recruitment and consent will be evaluated based on the consent rate and any barriers identified. The intervention will be assessed for acceptability by the participants based on participation levels, compliance, and retention. The results from self-reports and questionnaires will be evaluated to determine patient compliance and assess the specific questions for comprehension by the participants as well as their suitability to provide the required data for evaluation. Several primary and secondary outcome measures are included in the pilot study, and specific metrics will be evaluated for reliability and feasibility to determine which are the most appropriate for evaluation of the intervention. These metrics will also be used for sample size estimations as required for performing a larger study.

The primary outcome measures for the pilot study are anthropometry, body composition, and cardiorespiratory exercise capacity. The secondary metrics include nutrition and physical activity outcomes, quality of life, cardiovascular and muscle function, and biochemical risk markers. Outcomes are assessed at baseline, 6 months, and 1 year at the Children’s Hospital, LHSC, and the Western University (UWO). These outcome measures will be exploratory.

**Fig. 1:**
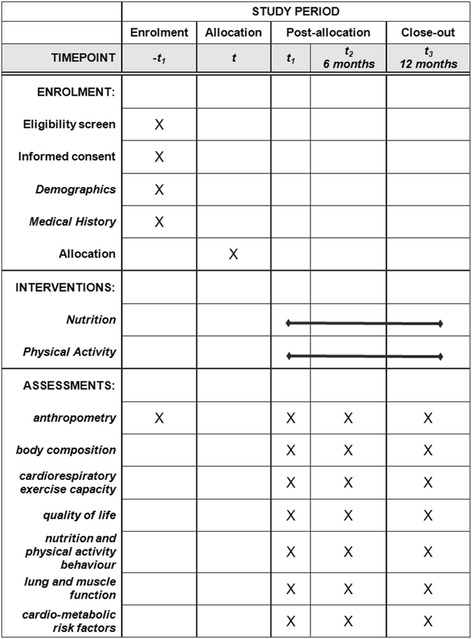
Schedule of enrolment, interventions, and assessments. The Standard Protocol Items: Recommendations for Interventional Trials (SPIRIT) diagram for the Smart Heart pilot study

This study is approved by the Western University Health Science Research Ethics Board (REB# 18843). Recruitment for the study was initiated in February 2013 and is currently ongoing. The Standard Protocol Items: Recommendations for Interventional Trials (SPIRIT) checklist for the Smart Heart study can be found in Additional file 1: Table S1. The schedule of enrolment, interventions, and assessments for the Smart Heart study can be found in Fig. 1.

#### Program staff

Nutrition and physical activity counseling are provided by a registered dietitian and fitness specialist, respectively. The investigating cardiologists supervise the exercise stress tests and perform the tilt table echocardiography. Designated clinic technologists and nurses perform the various medical procedures in hospital (i.e., electrocardiography [ECG], echocardiography, blood work) with the assistance of regular clinic administrative staff. Research blood work is analyzed at the Translational Research Centre lab at LHSC. A trained research assistant guides participants through procedures and performs additional tests, such as peripheral arterial tomography (PAT) and jumping plate assessments. This pilot study also collaborates with designated individuals for dual-energy X-ray absorptiometry (DXA), exercise stress testing, spirometry, accelerometry, and waist and hip circumference measures. A research coordinator assists with program administrative duties and data management. Program software was developed in collaboration with Department of Electrical and Computer Engineering at UWO.

#### Procedure

**Fig. 2:**
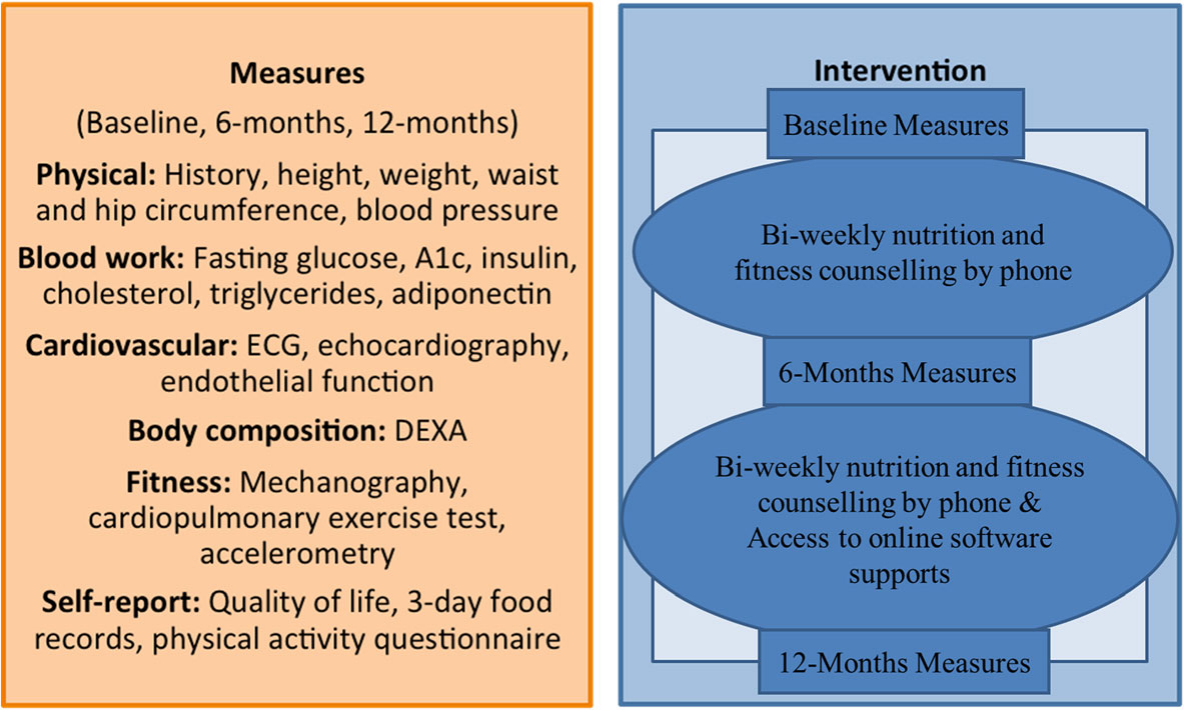
Outline of the pilot study procedure from baseline to 12-month measures. The Smart Heart Trial included three assessments and spanned 12 months. The physical, metabolic, cardiovascular, and body composition outcome measures were taken for each subject upon study entry at baseline and at follow-up after 6 and 12 months. Nutrition and fitness counseling was performed by mobile phone once per week, with the nutrition and fitness counseling support alternating weeks (i.e., 25 counseling sessions for each for a total of 50 sessions)

Commencing the study, participants undergo the following testing at baseline: height, weight, waist and hip circumference measures, blood pressure, blood work, electrocardiography, echocardiography, body composition (iDXA), lung function, cardiorespiratory exercise tests, and physical activity behavior (accelerometry). In addition, participants provide completed 3-day food records, quality of life, and physical activity questionnaires. This is repeated at 6-month and 1-year follow-up. Outline of the pilot study procedure from baseline to 12-month measures are summarized in Fig. 2.

#### Lifestyle intervention

During the initial hospital visit, participants are provided with a complimentary smartphone and one-year mobile plan. Participants without Wi-Fi at home are also given a limited data plan. The structured lifestyle intervention involves alternating weekly phone calls with two health coaches: a registered dietitian and a fitness specialist. A total of 50 phone calls (25 nutrition-related and 25 physical activity-related) are delivered over the one-year study. Phone counseling sessions are up to 30 min in duration. The duration of the call depends on the depth of material being covered and participant engagement. The phone calls are prescheduled for the year, taking place at a consistent evening time and day of the week. However, participants and coaches are encouraged to reschedule calls as needed between themselves. When appropriate, weekly counseling sessions are supplemented with relevant resources that are provided to patients via email or regular mail. After 6 months in the program, participants are introduced to supporting program-specific software that they can access online, as an additional way to interact with the program, set goals, and record their progress.

### Nutrition counseling

Client and family-centered nutrition counseling includes an initial nutrition assessment and 24 subsequent conversations (i.e., phone calls) using motivational interviewing techniques intended to increase nutrition knowledge or skills and improve nutrition and eating behaviors. Standard nutrition education is provided, as deemed beneficial by the coach and participant (Table 1). If a participant has a particular nutrition concern or interest (e.g., lactose intolerance, sports nutrition), this education is also provided. Participants are encouraged to set SMART goals at the end of each session, which are followed up 2 weeks later. Nutrition and eating behavior is re-evaluated on a regular basis through 3-day food records obtained at baseline, 6 and 12 months, and supplemented with 24-h diet recalls by phone at 3 and 9 months. In the latter half of the program, participants are provided with optional family nutrition challenges (monthly) and eating behavior questionnaires (4 in total) to stimulate further family engagement and self-monitoring behaviors.

**Table 1:** Nutrition and fitness program topics typically covered by time period for each participant

Time period	Program topics	
	Nutrition	Physical activity
Part 1: 0–6 months	Canada’s Food Guide, balanced meals, moderation, the division of responsibility in feeding, meal planning, healthier meal and drink ideas, grocery shopping, eating out, healthy drinks, label reading. *Family engagement*: bi-weekly resources *Evaluation*: Every 3 months (food records or diet recall)	Canada’s Physical Activity and Sedentary Behavior Guidelines, benefits of physical activity, sedentary behavior, interconnection of lifestyle behaviors and environment (sleep, activity, school), behavior change strategies. *Evaluation*: bi-weekly (physical activity recall)
Part 2: 6–12 months *Daily use of program-specific Internet-based application recommended*	Packing lunches, focused eating (satiety cues, emotional eating), eating out, sugar-sweetened drinks, family meals, eating breakfast, meal planning, label reading, increasing vegetables and fruit *Family engagement*: Monthly nutrition challenges *Evaluation*: every 3 months (food records or diet recall), eating habits questionnaires (4 in total)	Challenging current physical activity levels and diversity of movement choices, sustaining motivation levels and incorporation of rewards. *Evaluation*: bi-weekly (physical activity recall)

### Physical activity counseling

Client and family-centered fitness counseling includes an initial fitness assessment and 24 subsequent conversations (i.e., phone calls) using motivational interviewing techniques intended to improve physical activity participation by gradually increasing the intensity, duration, frequency, and diversity of movement endeavors (Table 1). Furthermore, participants’ specific physical or sedentary activity-related concerns (e.g., feasible exercises at home, exercise modifications, injury recovery) are also addressed. Participants are encouraged to set SMART short- and long-term goals, and strategies to achieve them, at the end of each session, which are followed up in 2 weeks’ time. Physical and sedentary activity levels are assessed bi-weekly.

### Attendance and contact

Attendance for phone counseling sessions is recorded as a proxy measure for participants’ compliance with the intervention. The preferred day and time for phone sessions is selected by participants and pre-scheduled upon enrolment in the study. Phone calls are initiated by the lifestyle coaches on an alternating, weekly basis, such that the participant will be in contact with one life coach each week. Reminder text messages are sent in advance of the phone appointments. Voicemail and text messages are utilized to re-establish contact in the case of a missed appointment.

### Special technology and programming

Smartphones are provided to participants at no cost. In general, recent Android-based models, such as LG and Samsung, as well as BlackBerry, are utilized. Telecommunication packages include unlimited local and long-distance calling and text messaging within Canada.

### Program-specific software

An Internet-based component of the pilot study was developed for participant use from a home computer or mobile device of choice to (a) provide participants with optional email addresses connecting children and youth directly with their health coaches, (b) provide a platform for accessing nutrition and physical activity resources by all family members, and, (c) in the latter half of the program, provide access to a tracking and goal-setting application for participants. Participants and parents provide consent for email communications upon enrolment in the program and may choose to use their existing email address, the email address provided by the study, or to communicate exclusively by phone and regular mail. The web-based application is initiated after 6 months in the program with the intention of extending participant engagement at a time point in which lifestyle interventions tend to plateau in weight loss outcomes [19, 20]. Self-monitoring and goal-setting are two components of successful pediatric obesity intervention programs [21]. The web server utilized for the study is ISQ Solutions Inc. For privacy and confidentiality of the participants, only non-identifying numerical data are collected and stored in a secure SQL DB format. User authentication is required and generic logins using alpha-numeric study ID’s are provided to each participant in order for researchers to make sense of the data collected. Participants are encouraged to log in to the web-based application daily to record activity and nutrition behaviors and to set goals for the following day (Table 2). Health coaches have administrative access to the application in order to follow and comment on each participant’s progress. The data collected on the web-server is used primarily to inform health coaches in their practice. Data are also used for evaluating the web application (patterns of use) and defining participant engagement in the program.

**Table 2:** Summary of web-based application content from a participant perspective—participants enter numerical values

Behavior tracking	Goal setting
1. Minutes of exercise logged today.	2. Minutes of exercise planned for tomorrow.
3. Minutes of screen time logged today.	4. Minutes of screen time planned for tomorrow.
5. Number of “treats” eaten today (e.g., chips, pop, candy).	6. Number of “treats” planned for tomorrow.
7. Servings of vegetables and fruit eaten today.	8. Servings of vegetables and fruit planned for tomorrow.

### Feasibility outcomes

#### Integrity of the study protocol

The number of patients eligible to participate in the study based on the exclusion and inclusion criteria will be used for evaluation. If fewer than 10 patients are eligible per month, the exclusion/inclusion criteria will be re-examined and/or the time frame for recruitment adjusted for future studies. A complete run through of the protocol will identify any critical unforeseen issues with equipment, data collection, metrics used, timing, scheduling, training, and procedures. Adjustments to the protocol logistics and/or processes will be made where appropriate so the protocol is efficient and appropriate to meet the goals of the study. Feedback elicited from the research assistants, nutritionist, fitness specialists, and participants will be used for evaluation. All instruments/protocols used for data collection will be required to collect acceptable data from ≥ 80% of the study participants.

#### Recruitment and consent

The recruitment and consent rate will be used for evaluation. The objective is to reach a goal of at least 10 patients eligible for recruitment per month with an overall consent rate of ≥ 70%. Any barriers to recruitment will be identified based on the inclusion/exclusion criteria and feedback from those eligible patients who declined to participate in the study as well as the physicians, both participating and not.

#### Acceptability of the intervention

The intervention will be evaluated for acceptability through assessment of patient participation, compliance, and retention. These metrics will be obtained from the patients’ weekly reports, attendance records, level of engagement with the dietician and fitness specialist (i.e., missed and/or rescheduled appointments, length of the telephone conversations), and the retention rate. A minimal retention rate of ≥ 70% with ≥ 80% compliance would be acceptable.

#### Self-reports and questionnaires

Patient compliance will be used to evaluate patients’ self-reports and questionnaires. For those unanswered, or poorly answered, question feedback will be elicited from patients to determine the reason for non-compliance and assess their comprehension of the questions. Any specific questions with ≥ 50% non-compliance will be re-evaluated for suitability and re-written for any future studies. At least 80% compliance overall would be considered acceptable.

#### Selection of the most appropriate outcome measures

To evaluate the impact of the intervention, numerous surrogate metrics for improved diet and physical activity will be measured. The standard deviation (SD) and the 80% upper confidence limit of the SD will be calculated and used for sample size estimation for each of the primary outcome measures [22]. Those outcome measures that estimate sample sizes exceeding the overall median estimate from all primary outcome measures by 75% will be re-evaluated for use in any future studies.

#### Sample size estimates

Sample size estimates will be calculated from each interval or ratio-based primary and secondary measures. The 80% upper confidence limit of the SD will be used as the measure of sample variability to estimate sample size with alpha = 0.05 and beta = 0.20.

### Primary outcome measures

#### Anthropometry

Body weight and height are measured using an electronic scale to the nearest 0.1 kg and wall-mounted stadiometer to the nearest 0.1 cm, respectively. Waist and hip circumference are measured with a flexible tape to the nearest 0.1 cm. Hip circumference is measured a minimum of twice around the largest part of the buttocks. Waist circumference is measured a minimum of twice at the tip of the iliac crest. For both waist and hip circumference, an average is taken of the first two measures with internal variation of ≤ 1 cm.

#### Body composition measurements

A trained technician uses dual X-ray absorptiometry (iDXA; General Electric-Lunar iDXA, Ames Medical iDXA; Prodigy, enCORE 2007 software version 11.40.004, Waukesha, WI) to measure fat mass, lean mass, percent body and android fat, visceral adipose tissue, and bone mineral content. Lunar iDXA has been previously validated [23].

#### Cardiorespiratory exercise capacity

Breath-by-breath data on the volume of oxygen uptake (VO_2_) and carbon dioxide production are collected during a maximal exercise graded treadmill test using a Cosmed Quark b^2^ indirect calorimetry metabolic system (Cosmed S.r.I, Rome, Italy). An electrocardiogram is simultaneously used to monitor heart rate. The goal is to determine the peak VO_2_ based on the respiratory exchange ratio (*R*) ≥ 1.05 [24].

### Secondary outcome measures

#### Nutrition

Participants complete 3-day food records, which are reviewed through a single, multi-question pass by a registered dietitian. Recipes and brand names are collected when possible. Portion estimation, rather than weighed portions, is used to limit respondent burden. Thorough instructions and a portion estimation guide are provided in advance. The registered dietitian manually tabulates average intake from four Canadian food groups (servings of vegetables and fruit, grain products, milk and alternatives, and meat and alternatives) and fluid. Average caloric intakes are estimated for total energy, calories from “other foods” (e.g., candy, snack foods, and condiments), and calories from “sugar-sweetened beverages.”

#### Physical activity

Physical activity is objectively assessed using the Actical® (Minimitter, Oregon), which is a small, water-resistant, omni-directional accelerometer. This device is sensitive to low frequency movements in the range of 0.5–3.2 Hz, which is the common range for human movement. Participants are asked to wear this device on the right hip during the waking hours (at least 12 h) for 7 days. Participants record a daily log of their Actical use, including times when the device is removed or replaced, and times of significant physical activity. The Actical has been previously validated for use in the pediatric population and those with CHD [25, 26]. Self-reported physical activity is also assessed using the Physical Activity Questionnaire for Older Children (PAQ-C, Version 2, 2009) [27]. This is a nine-item, 7-day, recall questionnaire for older children that assesses general moderate-to-vigorous physical activity during the school year. The PAQ-C is previously validated for use in children aged 8–14 years [28]. Participants and parents are also asked if the parents have ever restricted the participant’s physical activity because of the heart condition.

#### Quality of life assessment

This study uses the Pediatric Quality of Life Inventory Generic Core Scales (PedsQL™, Version 4.0, ©1998 JW Varni) parent and child (8–12 years) or parent and teen (13–18 years) reports to evaluate physical and psychosocial (emotional, social, and school) functioning. PedsQL™ Generic Core Scales are previously validated for use in children with heart disease [29].

#### Assessment of muscle function

**Fig. 3:**
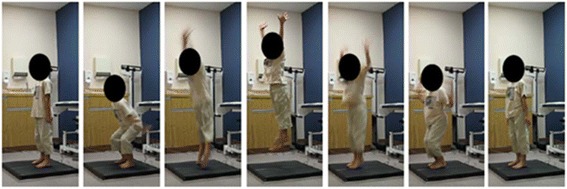
Eight-year-old boy performing a two-leg jump on the Leonardo Mechanograph® platform

Mechanography is a relatively new tool for assessing muscle function in children. This study used the Leonardo Mechanograph® Ground Reaction Force Plate (Novotec Medical Inc., Pforzheim, Germany) and proprietary software (GRFP Research Edition® software, version 4.2-b05.53-RES.). The device is a quadratic platform divided into two sections that can measure the vertically applied forces from the right and left lower limbs separately (Fig. 3). Signals from the four plate sensors are recorded by a portable computer using a 2.0 USB connection. Depending on the test performed, the software captures force data and derives acceleration, power, energy, speed, and jumping height. Reference values have been generated for children and adolescents using the Leonardo Mechanograph® platform, and reproducibility has been ascertained in healthy populations of children [30].

#### Electrocardiographic measurements

A standard 12-lead ECG is obtained by a qualified technician on supine participants who have been resting in position for at least 5 min. The ECG markers measured and analyzed include criteria for left or right ventricular hypertrophy, heart rate, QRS duration, and PR and QT intervals.

#### Transthoracic echocardiographic measurements

Following a modified functional protocol based on the American Society of Echocardiography guidelines and using commercially available machines (Vivid S6 or Vivid 9, GE Medical Systems, Milwaukee, WI, USA), parasternal long axis, parasternal short axis, and apical 4, 2, and 3 chamber images are acquired to measure M-Mode, two-dimensional data are used to obtain left ventricular volumes, ejection fraction, and strain analysis, and Doppler and tissue Doppler are used to measure additional systolic and diastolic parameters, while participants are lying supine [31]. Left ventricular strain is analyzed off-line using a commercially available software analysis (EchoPac, Version 7.1, GE Medical Systems, Milwaukee, WI, USA), following published guidelines [32].

#### Endothelial function

Endothelial vasodilator function, as an indicator of the earliest signs of cardiovascular disease, is assessed using peripheral arterial tonometry with the Endo-PAT 2000 (Itamar Medical Ltd., Caesarea, Israel) [33]. The fasting participant lies supine without movement for 15 min while the reactive hyperemia index is measured by finger-tip plethysmography following occlusion of brachial artery blood flow for 5 min in one arm, compared to the non-occluded contra-lateral arm. The reactive hyperemia PAT index (RH-PAT) is then calculated using an automated algorithm. RH-PAT and log-transformed values for each participant at the three intervention time points are entered into a database for statistical analysis. A lower value is indicative of a greater degree of cardiovascular disease and other conditions associated with impaired endothelial function or cardiovascular disease risk. Dietary and exercise intervention can improve endothelial dysfunction in obese children [34].

#### Laboratory measurements

Blood is drawn from patients who have been asked to fast for at least 10 h. The blood work includes lipid profile, electrolytes, creatinine, urea, fasting glucose, A1C, serum insulin, and adiponectin, a serum protein inversely associated with metabolic syndrome and insulin resistance [35]. In collaboration with the Translational Research Centre, the serum is stored in repository at − 85 °C until further analysis.

#### Participant feedback

When exiting the study, participants, and their accompanying parent or caregiver, are asked to complete a program evaluation form with ratings and comments on the life coaches, the technology, and the participant experience, including suggestions for improving the intervention program. Comments and scores for each question of the evaluation form are entered into a database. Anonymous metadata are used by staff and investigators for continuous quality improvement and decision-making. This qualitative information is also valuable for evaluating the research program from a participant and family perspective.

### Sample size for the pilot and feasibility study and statistical analyses

A relatively small sample size (*n* = 40) was selected to test the feasibility of the intervention. A power calculation was not used as we were unable to determine the variability and expected difference in our primary outcome measures for our study population. As such, the results from the current study will provide the required information to perform power calculations for future studies.

The mean, SD, 80% confidence intervals, and 95% confidence intervals (95% CI) will be calculated and reported for all ratio and interval-based outcome measures, for each of the three time points and overall. The following hypothesis testing will also be performed, but purely as an exploratory exercise and as a component of the pilot. Temporal changes in primary and secondary outcome measures will be assessed using a repeated measures analysis of variance. Specifically, data will be analyzed using analysis of variance (ANOVA) and covariance (ACNOVA) with Bonferroni post hoc comparisons with IBM SPSS Statistics for Windows, version 24 (IBM, USA). Participants with missing data may be excluded from analysis if relevant. These data will be critical for informing future large-scale, controlled trials and establishing variances for the outcome metrics for sample size calculations.

## Discussion

In summary, this article has provided a rationale for the development of the intervention and an explanation of its study design and methods. This article has presented all of the information needed to develop and deliver a similar lifestyle intervention program, including making use of mobile technology as well as the study design, intervention components, and recruitment and measurement procedures. Although this feasibility study is ongoing, with an estimated completion date of the end of 2017, anecdotal reports from children and parents have been largely positive.

Despite the high completion rates, owing to the minimal burden on participating families, the following challenges were identified for future consideration of similarly designed studies: study compliance, technology, and measuring fitness and nutrition behavior.

The study is designed to mitigate certain potential compliance issues. First, adherence to the weekly telephone sessions may be challenging. For this reason, participants receive reminder text messages prior to their scheduled appointment times, which are rescheduled when necessary. The online supporting software may also be underutilized. To minimize this, participants are provided with in-person instruction on the software and are reminded to use the software through text messages and during counseling sessions.

The development of the online software required significantly more time than was originally planned, due, in part, to privacy reviews requested by the institutional REB. Researchers should temporally budget for software development and include respective institutional and governing research bodies early in the design process.

Quantifying physical activity is difficult because it is a multidimensional behavior; calculating the intensity, duration, frequency, and type with an all-encompassing tool is a challenge. Thus, accelerometers were chosen as an objective method of characterizing physical activity for this research project. Similarly, a variety of subjective and objective methods exist to characterize nutrition behavior. In this case, 3-day food records were chosen as the best approach to collect a variety of information about current participant intake and eating habits (e.g., timing of eating episodes, portion sizes, breakfast habits), which is useful for immediate interpretation by the registered dietitian in providing ongoing guidance to participants, with minimal participant burden.

A specific strategy for obesity management among pediatric cardiology patients, such as nutrition and physical activity guidelines and/or programs for at-risk patients, is lacking. The results from this pilot study will be made available with the intention to further knowledge in the management of pediatric obesity in CHD patients and in the application of smart mobile technology to pediatric health care programs in general. In the meantime, it is anticipated that the matters discussed in this article will inform researchers and health professionals developing related trials and programs.

## Additional file

Additional file 1: SPIRIT 2013 Checklist: Recommended items to address in a clinical trial protocol and related documents*. (DOC 121 kb)

## Abbreviations

BMI: Body mass index
CHD: Congenital heart disease
DXA: Dual-energy X-ray absorptiometry
ECG: Electrocardiography
GRFP: Ground reaction force plate
LHSC: London Health Sciences Centre
PAQ-C: Physical Activity Questionnaire for Older Children
PAT: Peripheral arterial tomography
PedsQL: Pediatric Quality of Life Inventory Generic Core Scales
REB: Research Ethics Board
UWO: University of Western Ontario
